# Hypoxia increases the risk of egg predation in a nest-guarding fish

**DOI:** 10.1098/rsos.160326

**Published:** 2016-08-31

**Authors:** Karin H. Olsson, Charlotta Kvarnemo, Maria Norevik Andrén, Therése Larsson

**Affiliations:** 1 National Institute of Aquatic Resources, Technical University of Denmark, Lyngby, Denmark; 2 Department of Biological and Environmental Sciences, University of Gothenburg, Gothenburg, Sweden

**Keywords:** parental care, clutch cannibalism, low oxygen, egg predation, nest defence

## Abstract

For fish with parental care, a nest should meet both the oxygenation needs of the eggs and help protect them against predators. While a small nest opening facilitates the latter, it impedes the former and vice versa. We investigated how the presence of potential egg predators in the form of shore crabs *Carcinus maenas* affects nest building, egg fanning, defensive displays and filial cannibalism of egg-guarding male sand gobies *Pomatoschistus minutus* under two levels of dissolved oxygen. In the high oxygen treatment, males retained their nest opening size in the presence of crabs, while males in low oxygen built large nest openings both in the absence and presence of crabs, despite the fact that crabs were more likely to successfully intrude into nests with large entrances. Males in low oxygen also fanned more. In the presence of crabs males increased their defensive displays, but while males in high oxygen reduced fanning, males in low oxygen did not. Filial cannibalism was unaffected by treatment. Sand gobies thus prioritize egg ventilation over the protection afforded by small nest openings under hypoxia and adopt defensive behaviour to avert predator attention, even though this does not fully offset the threat from the egg predators.

## 1. Introduction

Many species construct nests that shelter eggs and young, allowing parents to control the microclimate and thus optimize the developmental conditions of the offspring [1–3]. However, with predation often being the main cause of breeding failure [4,5], reproductive success is also closely tied to nest defence, imposing potentially conflicting demands on the architecture of the nest. Arguably, most studies on the importance of nest characteristics have focused on birds, but the importance of nest attributes to the breeding success of species of fish, insects and spiders is becoming increasingly better understood.

Nest defence strategies can be either stealthy, seeking to avoid the attention of predators through camouflage and the adoption of inconspicuous behaviour, or active in the form of mobbing, distracting or outright attacks for the purpose of deterring or misleading the potential predator. Multiple studies have demonstrated the link between nest crypsis and nest survival, mostly in birds (e.g. [6–9]) but there is also ample evidence on the importance of nest camouflage in fish [10,11]. Furthermore, the apparent density of predators may affect the nest-building efforts. For instance, the presence of predators caused three-spined sticklebacks *Gasterosteus aculeatus* to nest in more densely vegetated spots compared with the usually preferred open sites [12], while both sand gobies *Pomatoschistus minutus* and common gobies *P. microps* were observed to increase the sand cover of their nest in the presence of a predator [11,13]. Alternatively, egg survival may be ensured by the presence and aggressive behaviour of the parent (e.g. [14–17]). The level of aggression may vary as the clutch approaches independence or over the course of the breeding season, affected by the reproductive value of the clutch and the breeding opportunities that remain to the parents [1,5,18]. Stealthy and active nest defence strategies may to some extent be mutually exclusive as aggression tends to be conspicuous and may reveal the location of the nest. Thus, if offspring vulnerability changes with age, parents may alternate between stealthy and active defence [19]. Guarding parents may also calibrate the intensity of their defence, for instance by escalating from warnings to outright attacks, depending on the type of predator and its proximity to the nest, presumably reflecting the likelihood that the exact location of the nest has been discovered [20–22].

When the nest has a roof or some other form of cover, the size of the nest opening is particularly relevant as this is the means by which a predator may gain access to the clutch. In general, larger nest openings have been shown to be associated with higher predation rates [5,9]. Conversely, the high breeding success of hornbills is often ascribed to the habit of the female to seal herself inside the nest behind a mud wall, although if that wall is breached the clutch is often lost [23–25]. The size of the nest entrance may also be specifically adapted to the ability of the guarder to defend it. For instance, both the blenny *Salarias alboguttatus* and soldier termites of the species *Reticulitermes speratus* use their bodies to completely block the nest entrance in the presence of a predator [26,27], while the nest entrance of the johnny darter *Etheostoma nigrum* is small enough to deny larger-bodied crayfish access [28]. Finally, the size of the nest entrance may be under sexual selection. When nests are energetically expensive to build (e.g. [29,30]), their appearance may be considered a sexual ornament [9]. For instance, female common gobies have been shown to prefer nests with small entrances [31]. Furthermore, smaller nest entrances may also facilitate defence against intrusions by conspecifics, such as competitors and parasitically spawning males [32,33]. In fact, male sand gobies have been shown to reduce the size of their nest entrance in the presence of sneaker males [34].

In aquatic habitats, nest-tending parents may face a trade-off between the advantages a well-concealed nest can offer and the need to ensure that the eggs are adequately oxygenated. Water carries less dissolved oxygen than does air, and hypoxia, i.e. a level of reduced dissolved oxygen that impairs behaviour or physiology of an organism, is not unusual [35]. Shallow, coastal habitats are particularly variable in terms of temperature, salinity, acidity and oxygen saturation [36], and increasing eutrophication makes hypoxia more frequent [37]. Insufficient oxygenation has been linked to retarded egg development, reduced hatching success, impaired larval condition, increased incidence of deformities and increased mortality in a range of fish species (e.g. [38–45]). In nest-building species of fish, oxygen concentration is often lower inside the nests than outside [46] and the construction of the nest can impede the flow of oxygen-rich water to the eggs [47]. Three-spined stickleback males modify the structure of the nest as embryos develop, presumably in response to increased oxygen demands [2]. Similarly, males of both sand gobies [48,49] and common gobies [11] increase the size of their nest openings in low oxygen conditions. Even so, in both species hypoxia is associated with delayed hatching and retarded egg development [49,50]. Moreover, faced with a trade-off between better egg defence through small entrances and better egg ventilation through large entrances, sand gobies opted for slightly reduced entrances [49] while the presence of an egg predator had no effect on nest entrance in common gobies [11].

Earlier work has assessed a variety of responses by nest-guarding males to visual and olfactory cues of the presence of a predator; however, we believe that none has attempted to assess the efficacy of these responses by staging a full-on confrontation between the guarder and an egg predator. Using egg-guarding male sand gobies, we investigated how hypoxia affects the trade-off between predator defence, in the form of active aggression and nest concealment versus egg ventilation through fanning and larger nest entrances. We also studied how these conditions affect egg mortality due to filial cannibalism and the probability of predators successfully intruding into the nests. This study builds upon the results obtained by Lissåker & Kvarnemo [49], which showed that sand goby males increased the size of their nest entrances in low oxygen, but reduced it again if an egg predator (a small tethered shore crab) was present. On the other hand, they found no effect of the presence of a predator on the fanning expenditure, guarding behaviour or full or partial clutch filial cannibalism. By comparison, in this study crabs were allowed to move freely in the tank, allowing us (i) to more closely mimic the situation in the wild and (ii) to study the actual, rather than merely the potential, threat of a predator and to assess the efficacy of the actions the guarding male may take to mitigate the danger to his nest.

## 2. Material and methods

### 2.1. Study species

The sand goby *P. minutus* is a small fish found in coastal waters in northern Europe where it breeds in shallow, sandy bays [51]. Both males and females are polygamous and spawn from April through June. The male builds a nest by excavating a hollow beneath an empty mussel shell which he then covers with sand. Through courtship displays including erect fins that emphasize an iridescent blue spot on the dorsal fin and a darkened anal fin, males attract females to spawn inside the nests (reviewed in [52]). After entering the nest, courtship behaviour also includes sound production [53,54], which is amplified by the amount of sand piled on top of the nest [55]. During spawning, females attach their eggs in a single layer to the ceiling of the nest after which the male alone cares for the eggs. Care lasts until the eggs hatch and includes maintaining and defending the nest against predators and fanning the eggs by fin beats using both anterior and posterior fins [52]. Predators are deterred by defensive displays, which include darkened and erect fins, sometimes followed by direct attacks, including pushes and bites. In the case of shore crabs *Carcinus maenas*, guarding males have been observed to target the eye stalks of the crabs during attacks (T.L. 2006, personal observation). A predator in the immediate vicinity of the nest entrance may cause the male to intensify the attacks while moving away from the nest, apparently luring away the predator (T.L. 2006, personal observation).

### 2.2. Experimental design

The study was conducted at Kristineberg Research Station (Sven Lovén Centre for Marine Sciences, 58°15′ N, 11°28′ E) by the Gullmar fjord on the Swedish west coast in May and June, 2006. Sand gobies were caught using a hand trawl in Bökevik bay near the station. After capture the fish were sexed and kept in separate storage aquaria (130 l, supplied by running seawater) in the laboratory for acclimatization at least 3–4 days. While in storage tanks, the fish were fed daily on chopped fresh mussel meat *Mytilus edulis*. The study used a two-by-two set-up consisting of a high oxygen treatment (100% dissolved O_2_) and a low oxygen treatment (35% dissolved O_2_). The level of dissolved oxygen in the low oxygen treatment is similar to naturally occurring bouts of hypoxia in the breeding habitat of sand gobies, which are tolerant to fluctuating oxygen levels [56]. The 35% level has been used before in similar studies where it was sufficient to elicit effects on behaviour without injuring the fish (e.g. [11,31,48,50]). Within each oxygen treatment, the experimental fish were either exposed to egg predators in the form of two shore crabs *C. maenas*, or not, serving as control. Shore crabs are common in the breeding habitat of sand gobies and prey upon goby eggs opportunistically. The experiment was conducted in 12 aquaria (20 l, supplied with running seawater), of which six were randomly assigned to each oxygen treatment. This set-up was used repeatedly, with new replicates started as previous ended. The individual aquaria used for high and low oxygen treatment remained the same over the whole study, whereas aquaria used for predator and predator-free replicates were changed throughout. Screens were affixed to the sides of all aquaria to prevent visual interactions between replicates. Each experimental aquarium was provided with a halved clay flowerpot to serve as a standardized nest site, a 4 cm layer of sand in which to burrow and hide, and an air stone to which either an air pump or a cylinder of nitrogen gas was connected, depending on oxygen treatment.

### 2.3. Oxygen treatment

At the beginning of the experiments, each male was measured to the nearest mm (mean ± s.e: 51.2 ± 1.2 mm, *n* = 60; length did not differ between oxygen or predator treatments, two-way ANOVA: oxygen: *F*_1,57_ = 0.58, *p* = 0.45, predator: *F*_1,57_ = 1.14, *p* = 0.29; interaction: *p* = 0.95), randomly assigned to a treatment and placed in an aquarium with a gravid female. Males that failed to start building a nest and females that failed to spawn in a built nest within 24 h were removed and replaced with new individuals. Shortly after spawning (usually in the early morning, as females tend to spawn during the night), the female was removed. The delay offered the male time to repair any damage of the nest entrance that sometimes occurs during spawning, before we measured its size. Assuming that the nest opening is elliptical in shape, we measured its height and width and calculated the size as area = *π* × height/2 × width/2. Since females lay their eggs in a single layer, the number of eggs can be estimated from the area occupied by the clutch. We picked up the flower pot and marked the outline of the clutch in pencil, then returned it to the aquarium, allowing the male to rebuild the nest. After the flowerpot was returned, the water flow was cut. In the high oxygen treatment, air was continuously bubbled into the water through an airstone, to maintain oxygen saturation. In the low oxygen treatment, the level of dissolved oxygen was gradually lowered to 35% over a period of 45 min by bubbling nitrogen gas into the water, gently stirring the water to lower oxygen saturation evenly in the aquarium, while monitoring oxygen saturation with an oxygen meter (Oxy Guard Handy Delta). The lids of the aquaria in the low oxygen treatments were covered in cling wrap to prevent oxygen fluctuations. All aquaria were kept at constant temperature (mean water temperature 14.4 ± 0.013°C) and temperature and oxygen levels were checked at least once a day with an oxygen meter. Oxygen levels in the low oxygen treatment were kept stable by bubbling additional nitrogen or oxygen gas into the water as needed. Particular care was taken to ensure stable oxygen levels in the predator treatment, as the addition of the crabs increased the oxygen consumption.

### 2.4. Predator treatment

Shore crabs were caught by hand or boat trawl in Bökevik Bay, where they are abundant. Prior observations of interactions between non-experimental fish and shore crabs suggested that small crabs were easily deterred by the fish. We thus chose crabs (carapace width mean ± s.e.: 39.3 ± 1.2 mm; crab carapace width did not differ between treatments, one-way ANOVA: *F*_1,28_ = 0.13, *p* = 0.73) just large enough to present a threat to the eggs, but not to the adults, assuming that the adult fish, which are nimble swimmers, would be able to avoid the crabs by abandoning the nest. Notably, no fish were injured, even though the confined aquarium environment might give crabs the advantage. The crabs were kept in storage aquaria (60 l) under the same conditions as the fish for acclimatization. Half of the replicates in the high and low oxygen treatments were randomly assigned to the predator and half to the control treatment. Following the initial nest and clutch examination, two crabs confined in a clear perforated plastic container were introduced into the aquaria assigned to the predator treatment, while aquaria in the control treatment received an empty container. After 24 h, the containers were removed and the crabs in the predator treatment were released. Similar to Lissåker & Kvarnemo [49], each replicate was observed visually for 15 min after release during which time the number of direct attacks was counted and the time the male allocated to fanning, defensive displays, nest building and care of eggs was recorded using a stopwatch. Only defensive displays (from now on referred to as ‘displays’) and fanning were observed with sufficient frequency to be further analysed. The intensity of fanning was estimated as the number of fin beats in three bouts of fanning, recorded using a counter, divided by the total duration of the three bouts. It was not possible to conduct blinded observations, since the treatment was visually evident (the presence of crabs and cling film). The crabs were left in the aquaria with the male during the entire trial period, which was set to 72 h. At 14.4°C, this corresponds to approximately one-third of the brood cycle [57]. At the end of the experiment, the nests were assessed for signs of a successful invasion by the crabs (crab inside nest or nest overturned or moved out of position) and the final egg area was noted in pencil. To estimate percentage egg loss, we used a method common in sand goby studies (e.g. [29,48,49]): We transferred the initial and final areas marked in the nest to paper, cut them out and weighed them to the nearest 0.001 g. Hence, any egg loss was estimated as the difference in weight.

A total of 62 replicates were obtained, 16 in the control treatment (i.e. without predator) with high oxygen, 15 in the control treatment with low oxygen, 15 in the predator treatment with high oxygen and 16 in the predator treatment with low oxygen. However, two males from the predator treatment in high oxygen cannibalized their whole clutch before the crabs were released and were excluded from further analysis, thus leaving us with 16, 15, 13 and 16 replicates. Sand gobies hide by burrowing in sand, and replicates were omitted from the behavioural analyses if the male could not be located and behaviour ascertained at any time during the 15 min. The remaining number of replicates for the display and fanning analyses were 11, 12, 10 and 13 and for the fanning intensity analysis 10, 11, 9 and 9. All data can be found in the electronic supplementary material.

### 2.5. Crab eating trial

To verify that crabs posed a similar threat to the eggs irrespective of the level of dissolved oxygen, a separate experiment was conducted to determine the effect of the oxygen level on the voracity of the crab. Crabs of similar size to those in the predator experiment (carapace width mean ± s.e.: 40.7 ± 1.02 mm, *n* = 40; crabs did not differ in size between treatments, one-way ANOVAs: carapace width: *F*_1,38_ = 0.45, *p* = 0.51; body mass: *F*_1,38_ = 0.30, *p* = 0.59) were left to fast for 1–3 days and randomly assigned to high or low oxygen treatment. Each crab was placed in an experimental aquarium (20 l) after which the water flow was cut and oxygen level was adjusted in the manner described above. The crab was allowed to acclimatize for at least 1 h. A large piece of fresh mussel meat was blotted dry, weighed to the nearest 0.01 g (College Mettler Toledo B502) and offered to the crab. The time it took for the crab to start feeding and the time it kept eating were recorded. If the crab stopped eating for more than two minutes or released the mussel, the trial was ended. The amount of meat consumed by the crab was calculated by blotting the leftovers dry and re-weighing them. After the experiment the crabs were released back into the bay. The level of dissolved oxygen did not affect the voracity, measured in gram mussel meat per second, of the crabs (one-way ANOVA: *F*_1,38_ = 0.012, *p* = 0.91), or the latency before they commenced feeding (Welch's ANOVA: *F*_1,23.28_ = 1.60, *p* = 0.22).

### 2.6. Statistics

All data were inspected and assessed for homoscedasticity using Hartley's *F*_max_ test. General linear model ANOVAs were used whenever applicable. Defensive displays were analysed using generalized linear models with a quasi-poisson error structure, while fanning intensity and mean fanning bout duration were analysed using separate Welch's ANOVAs for unequal variances. Significance level was set at *p* = 0.05 and non-significant interactions were removed from final models. Logistic regressions were used to test the effect of multiple predictors on a categorical-dependent variable (nest intruded or not) while categorical data on males fanning or not, crab intrusion and filial cannibalism were analysed using *χ*^2^-test or Fisher's exact test. All analyses were performed using R v. 3.2.1 software.

## 3. Results

### 3.1. Nest opening

**Figure 1.**
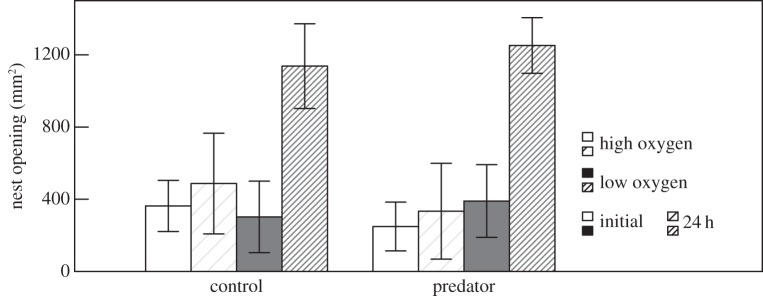
Nest opening size (mean ± s.e., mm^2^) initially and 24 h later in high and low oxygen for the predator treatment (nests built by egg-tending males housed with egg predators) and the control (without egg predators).

There was no difference in the initial nest opening size between treatments (two-way ANOVA, oxygen: *F*_1,57_ = 0.16, *p* = 0.69, predator: *F*_1,57_ = 0.011, *p* = 0.92; interaction: *p* = 0.26). However, after 24 h, the nest opening size was significantly larger in the low oxygen treatment than in the high oxygen treatment, while there was no effect of predator presence (two-way ANOVA, oxygen: *F*_1,57_ = 41.44, *p* = 2.74 × 10^−8^, predator: *F*_1,57_ = 0.014, *p* = 0.91; interaction: *p* = 0.27; figure 1).

### 3.2. Fanning occurrence, intensity and duration

**Figure 2.**
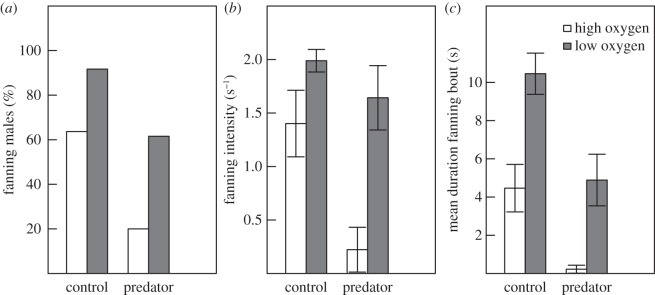
Fanning in high and low oxygen for the predator treatment (males housed with predators) and the control (males housed without predators). In (*a*) per cent of males observed fanning, in (*b*) fanning intensity (mean ± s.e., s^−1^) and (*c*) duration of fanning bout (mean ± s.e., s, based on mean duration over three bouts for each male).

Both oxygen level and predator presence had a significant effect on whether the male was observed fanning. More males fanned in the low compared with the high oxygen treatment, and more males fanned in the absence, compared with the presence, of a predator (*χ*^2^-test: *χ*^2^ = 11.83, d.f. = 3, *p* = 0.008, figure 2*a*). The effect of a predator on fanning intensity differed depending on the oxygen treatment. In the high oxygen treatment, it was significantly lower in the presence compared with the absence of an egg predator (Welch's ANOVA: *F*_1,15.82_ = 9.41, *p* = 0.0075) while in the low oxygen treatment the rate remained high also in the presence of an egg predator (Welch's ANOVA: *F*_1,9.62_ = 1.07, *p* = 0.33; figure 2*b*). The mean duration of the fanning bouts was significantly longer in low oxygen compared to high oxygen in both the control treatment (Welch's ANOVA: *F*_1,17.96_ = 13.57, *p* = 0.0017) as well as in the predator treatment (Welch's ANOVA: *F*_1,8.38_ = 10.33, *p* = 0.012). Overall, they were significantly shorter in the predator treatment compared to the control (Welch's ANOVA: *F*_1,36.90_ = 13.55, *p*-value = 0.00074; figure 2*c*).

### 3.3. Defensive display behaviour

**Figure 3.**
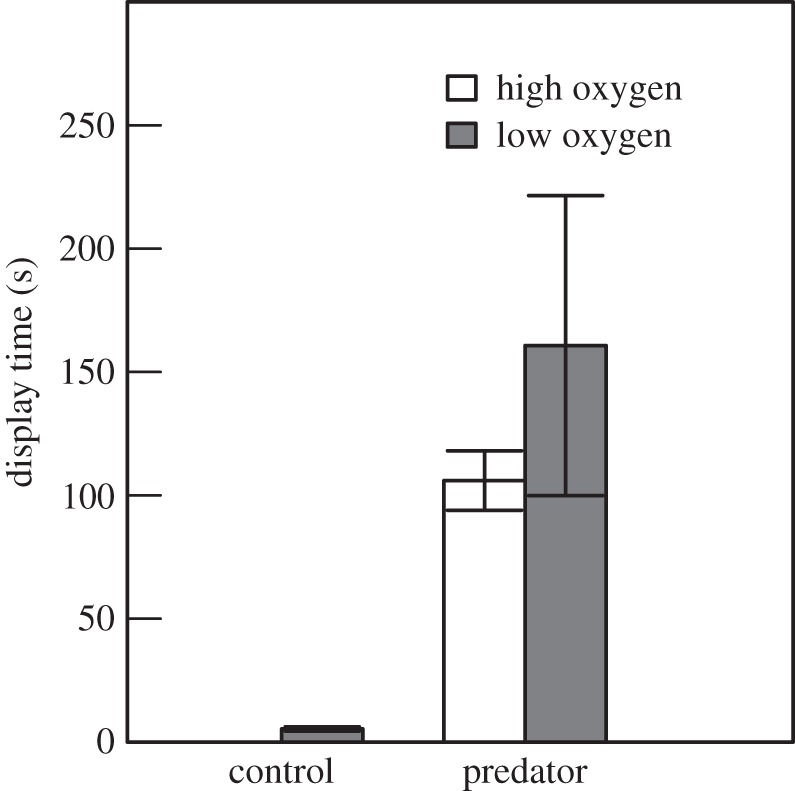
Effect of oxygen treatment on the amount of time males were observed displaying (mean ± s.e., s). Total observation time 900 s (15 min).

Males displayed significantly longer in the predator group compared to the control group, whereas oxygen level had no effect (GLM, predator: *t*_1,43_ = 2.72, *p* = 0.009, oxygen: *t*_1,43_ = 1.06, *p* = 0.29; interaction: *p* = 0.995; figure 3).

### 3.4. Nest intrusions

**Figure 4.**
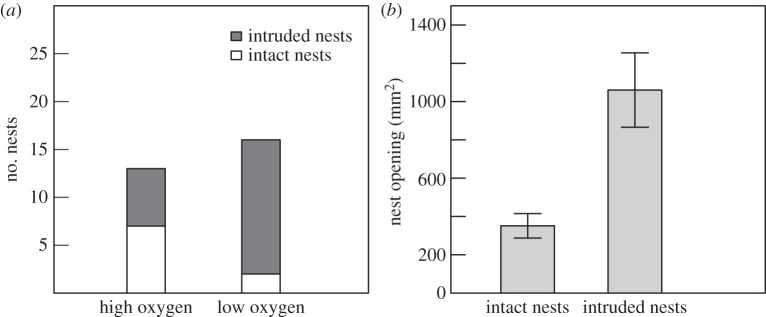
The effect of (*a*) oxygen treatment and (*b*) nest opening size (mean ± s.e., mm^2^) of nests left intact and intruded by crabs.

Analysis of nest intrusions was limited to the predator group. More crabs invaded nests in the low oxygen treatment than in the high (Fisher's exact test: *p* = 0.041, *n* = 29; figure 4*a*). Across both oxygen treatments, crabs were significantly less likely to intrude nests that 24 h into the trial had a small nest entrance, but the time spent displaying had no effect (logistic regression: table 1; figure 4*b*).

**Table 1.** Logistic regression model parameters (coefficient estimates, standard error, Wald *z* statistic and *p*-value) for effect of nest opening size after 24 h and display time on whether the nest was intruded by a crab or not. The interaction was non-significant (*p* = 0.60) and, therefore, removed.

	coefficients			
	estimate	s.e.	Wald *z* value	*p*-value (>|*z*|)
intercept	−1.60	1.03	−1.55	0.12
nest opening size	0.0031	0.0014	2.21	0.03
display time	0.02	0.01	1.21	0.23

### 3.5. Filial cannibalism and egg predation

To avoid conflating filial cannibalism with crab predation of eggs, only data from the control group were used to examine filial cannibalism. Only 3 of 31 males consumed the whole clutch, and all 3 came from the high oxygen treatment. In addition, eight males in the high oxygen treatment and four males in the low oxygen treatment consumed part of the clutch. However, neither full nor partial clutch cannibalism differed significantly between the oxygen treatments (full clutch cannibalism: Fisher's exact test: *p* = 0.23, *n* = 31; partial clutch cannibalism: *χ*^2^-test: *χ*^2^ = 0.93, *p* = 0.34). In the predation treatment, nests in which some egg loss could be recorded (irrespective of whether the source was filial cannibalism or nest intrusions) totalled 8 in the high oxygen treatment and 12 in the low oxygen treatment. Thus, oxygen treatment had no significant effect on the fraction of nests with egg loss (one-way ANOVA, *F*_1,27_ = 2.09, *p* = 0.16).

## 4. Discussion

Parental care is often a balance act between meeting conflicting needs of the offspring. For nest-guarding fish, a small nest opening helps protect the eggs against predators, but it also impedes oxygenation of the developing eggs. Investigating these trade-offs between oxygenation and defence of eggs, we found that hypoxia increases the risk of egg predation in sand gobies. Essentially, this result arose because parental males increased the size of the nest opening under hypoxia and maintained the enlarged entrances even in the presence of an egg predator, even though nests with larger openings were significantly more likely to be depredated. Instead, males displayed more in the presence of egg predators irrespective of oxygen level, which suggests that they rely on behavioural defence when a physical barrier in terms of a small nest opening becomes too costly. Nevertheless, this behavioural defence appears inadequate, at least in the aquarium setting, as more time spent displaying did not reduce the risk of nest intrusion. Thus, these results indicate that males treat insufficient oxygen supply as a greater threat than egg predation. Below we discuss the details of these results one by one.

### 4.1. Nest building

Male sand gobies experiencing hypoxia responded by increasing the size of their nest entrances, most likely to facilitate water exchange. This is in line with the findings in previous studies on nest-guarding fish [11,48–50]. Unexpectedly, in the current study the presence of a predator did not affect the size of the nest opening, irrespective of the oxygen level. Earlier studies have used restrained egg predators and have obtained mixed results. Specifically, a study on sand gobies found that males increased their nest openings in hypoxic conditions but decreased them again in the presence of a tethered egg predator [49], while a study on common gobies failed to find any effect of caged predators on the size of the nest entrance even in hypoxic conditions [11]. This suggests that the trade-off between nest defence and egg oxygenation is less straightforward than simply the size of the nest entrance.

What might prevent males from decreasing the nest opening in the presence of a predator, as found in the current study? It is unlikely that crabs are perceived to pose a lesser threat under hypoxic conditions, since oxygen level did not affect the appetite of the crabs, and compared with the previous studies, we used larger, more numerous and unrestrained crabs, which presumably pose a more palpable threat to the clutch. However, hypoxia may discourage nest building for several reasons. Physical activity is more energetically demanding under hypoxia ([48,50,58] but see [49]), and if the guarder has to prioritize some activities over others nest building may divert energy and attention from more critical activities such as egg ventilation and vigilance against predators. Moreover, even if the oxygen levels were checked frequently, the presence of crabs increased the oxygen consumption, which may have further deterred the males from expending energy on nest building, which also would make ventilation harder.

Even so, this does not explain why males in normoxia did not reduce the size of their nest entrances in the presence of egg predators. If nest defence relies on a combination of nest camouflage and direct attack [11] males may opt for active defence to repel the crabs, despite the lower success of this strategy. On the other hand, if guarders rely on stealth, they would be expected to behave as inconspicuously as possible, and it is possible that the presence of predators *per se* discourages prominent behaviour such as nest building [59,60]. Although there is little evidence that nest building itself attracts predators, it is a common observation that smaller birds are wary when building their nests and cease nest-building activities when potential predators are present [9]. Furthermore, we noted that if fish were inside the nest when the crabs were released they tended to remain there, whereas if fish were outside they generally did not enter the nest until the crabs had settled down away from the nest opening, which would support the stealth hypothesis. Reducing the nest entrance may, therefore, be just one of several different strategies available to a guarding male.

### 4.2. Nest ventilation and defensive displays

We found that males fanned more in low oxygen, which is consistent with the observation that fanning increases the flow of water through the nest [46] and would, therefore, be of greater importance during bouts of hypoxia. However, the presence of egg predators caused a reduction in the fraction of males that fanned their eggs at all and the mean duration of the fanning bouts. It also caused a reduction in the fanning intensity of males in the high oxygen treatment, but not of males in the low oxygen treatment. If nest defence primarily relies on stealth, the close proximity of egg predators would be expected to discourage males from conspicuous behaviour such that the presence of egg predators induces a slightly more cautious behaviour.

This is to some extent contradicted by the fact that males displayed more in the presence of egg predators, irrespective of oxygen level. If displays are largely motivated by the increased danger to the nest incurred by the larger nest openings, males in low oxygen would be expected to display even more, which was not the case. Thus, our results suggest that defensive displays are not calibrated by threat level or the cost of activity under hypoxia. However, since the presence of predators caused both the fraction of males that fanned the eggs and the duration of the fanning bouts to decrease but not the fanning intensity, it is possible that the activities are constrained in terms of time rather than energy. Similarly, other studies have found a trade-off between oxygenation and defence, as the time allocated to guarding correlated negatively with the time allocated to fanning, with low oxygen tilting the balance in favour of fanning [49], or diverting time from ventilation to attacks when egg predators were present [11].

### 4.3. Egg mortality from predation and filial cannibalism

Theory predicts that males may compensate an elevated cost of care caused by additional energy expenditures, for example, due to increased fanning in hypoxia, by a greater degree of filial cannibalism [61,62]. Poor somatic condition has been related to filial cannibalism in other fishes [63,64]. However, oxygen level did not affect the incidence of full clutch or partial clutch cannibalism in the predator-free treatment. The same was true for crabs, namely that their appetite for goby eggs was unaffected by oxygen treatment. Together, these results suggest that the increased occurrence of egg loss in the predator treatment was caused by the crabs rather than an increased cannibalism by males. That said, increased display efforts in the presence of crabs may also cause additional energy expenditure, which may induce a greater degree of filial cannibalism.

Larger nest entrances were found to significantly contribute to the risk of the nest being invaded by crabs, while defensive displays had no effect. Such a link between the size of the nest opening and incidents of predation has been demonstrated in both birds and fish (e.g. [5,28]), supporting the hypothesis that nest entrance is a key defensive aspect of nest architecture. Even so, our results suggest that fish do not prioritize nest entrance reduction, even in the presence of a predator. Instead, they attempt to counter the threat of predation by behavioural tactics such as defensive displays, which fail to fully offset the danger. Importantly, our study also demonstrates that environmental factors can affect the interplay between parents and egg predators. Here, hypoxia-induced enlarged nest openings clearly increase the risk of egg predation, thus shifting the advantage to egg predators.

These results were obtained using captive animals in the confined setting of experimental aquaria, and care should, therefore, be exercised when considering the effect of hypoxia on wild individuals. For instance, the behaviour of predators in captivity may be affected by the lack of alternative sources of food. However, although alternative sources of food may decrease the absolute risk to a nest, it does not contradict our finding that hypoxia increases the relative vulnerability of a nest. It may also be interesting to consider which particular aspect of nest entrance size might facilitate predator intrusion. The size of the nest entrance affects the visual appearance of the nest, but it is also possible that it influences its chemical signature. The foraging ecology of many crustaceans has been shown to be strongly reliant on olfactory cues (e.g. [65–68]), inviting speculation that small entrances may minimize the emission of such cues (so-called kairomones). On the other hand, the importance of vision appears to be habitat-dependent, such that species occupying flat and shallow environments have more well-developed eye stalks than species inhabiting more topographically complex environment [69]. On the basis of eye morphology, it is reasonable to assume that the shore crabs used in our study also rely on vision. Thus, while we find that the size of the nest entrance affects predation risk, how it does so is a topic for future studies.

In conclusion, male sand gobies prioritize egg ventilation over nest crypsis, which compels them to increase the size of the nest entrance under hypoxia. However, more exposed nests are at greater risk of intrusion by egg predators such as crabs, and males are unable to fully compensate for this additional threat through alternative anti-predator tactics. Hypoxia thus increases the risk of nest depredation and thereby lost reproduction.

## Supplementary Material

1 file with input data on size of fish and crabs, size of nest opening, cannibalism data and behavioural data, used in the analyses.

## Data accessibility

All data can be found in the electronic supplementary material.
