# Correction to ‘Hypoxia increases the risk of egg predation in a nest-guarding fish'

**DOI:** 10.1098/rsos.160907

**Published:** 2016-12-14

**Authors:** Karin H. Olsson, Maria Norevik Andrén, Therése Larsson, Charlotta Kvarnemo

The authorship order was presented incorrectly in the paper. The correct order should be Karin H. Olsson, Maria Norevik Andrén, Therése Larsson and Charlotta Kvarnemo.

