# Supplementary material for: Hypoxia increases the risk of egg predation in a nest-guarding fish
Source: R Soc Open Sci. 2016 Aug 31;3(8):160326. doi: 10.1098/rsos.160326 (PMC5108961; doi:10.1098/rsos.160326)
Supplement: 1 file with input data on size of fish and crabs, size of nest opening, cannibalism data and behavioural data, used in the analyses. [file rsos160326supp1.pdf]

Table 1:By experimental treatment; data on fish and crab length, nest invasion and egg survival, and nest opening size after 1 h and 24 h.

Oxygen treatment (High/Low)	Predator treatment (Control/Predator)	Fish length (mm)	Crab length (1) (mm)	Crab length (2) (mm)	Invasion (y/n)	Eggs left (%)	Nest opening (1 h) (mm^2)	Nest opening (24 h) (mm^2)
H	C	56				100	196.3	117.8
H	C	51				99	58.9	19.6
H	C	59				75	157.1	117.8
H	C	55				0	196.3	78.5
H	C	55				100	117.8	117.8
H	C	49				60	981.7	157.1
H	C	48				100	157.1	196.3
H	C	50				100	117.8	196.3
H	C	48				3	157.1	78.5
H	C	50				50	392.7	117.8
H	C	46				100	549.8	628.3
H	C	53				0	471.2	1413.7
H	C	55				98	883.6	314.2
H	C	48				0	471.2	1413.7
H	C	51				80	706.9	1413.7
H	C	48				45	196.3	1413.7
H	P	52	43	37	n	100	353.4	19.6
H	P	49	35	37	n	100	353.4	19.6
H	P	50	40	38	n	100	58.9	39.3
H	P	46	38	38	n	100	78.5	39.3
H	P	50	35	35	n	100	58.9	78.5
H	P	53	37	45	y	10	157.1	19.6
H	P	44	40	37	n	90	117.8	19.6
H	P	50	41	35	n	66	39.3	1079.9
H	P	49	37	40	y	0	294.5	78.5
H	P	53	41	40	y	0	196.3	196.3
H	P	50	45	39	y	0	39.3	353.4
H	P	52	41	43	y	60	785.4	1413.7
H	P	53	43	38	y	0	706.9	981.7
L	C	54				100	19.6	1079.9
L	C	48				100	117.8	196.3
L	C	53				100	196.3	196.3
L	C	57				100	19.6	471.2
L	C	50				75	39.3	981.7
L	C	49				100	98.2	1413.7
L	C	43				100	98.2	1413.7
L	C	60				100	19.6	1413.7
L	C	50				100	39.3	1413.7
L	C	53				20	294.5	1413.7
L	C	53				100	471.2	1413.7
L	C	53				98	353.4	1413.7
L	C	50				100	471.2	1413.7
L	C	64				98	883.6	1413.7
L	C	49				100	1413.7	1413.7
L	P	48	43	35	n	100	628.3	785.4
L	P	48	37	34	y	100	98.2	785.4
L	P	51	39	37	y	0	19.6	1413.7
L	P	70	45	42	y	0	78.5	1413.7
L	P	49	35	37	y	0	157.1	1413.7
L	P	52	40	43	y	100	98.2	1413.7
L	P	47	38	41	y	100	196.3	1413.7

L	P	49	43	39	y	0	196.3	1413.7
L	P	40	42	40	y	0	785.4	1413.7
L	P	57	37	42	n	85	785.4	1079.9
L	P	53	44	42	y	10	1413.7	1413.7
L	P	51	35	35	y	0	19.6	412.3
L	P	43	35	45	y	0	78.5	1413.7
L	P	50	37	39	y	0	353.4	1413.7
L	P	53	45	45	y	0	353.4	1413.7
L	P	54	38	35	y	0	981.7	1413.7

Table 2: By treatment, time males spent fanning and displaying, as well as fanning intensity and mean duration of a fanning bout, during 900 s observation (15 min).

Oxygen treatment (High/Low)	Predator treatment (Control/Predator)	Time fanning (s)	Time displaying (s)	Fanning intensity (s^{-1})	Mean fanning bout duration (s)
H	C	0	0		
H	C	237	0	1.83	11.33
H	C	0	0	0.00	0.00
H	C	0	0	0.00	0.00
H	C	110	0	2.10	5.67
H	C	225	0	1.87	10.00
H	C	35	0	1.70	4.67
H	C	85	0	2.54	5.33
H	C	32	0	1.97	4.33
H	C	45	0	2.00	3.33
H	C	0	0	0.00	0.00
H	P	0	28		
H	P	0	366	0.00	0.00
H	P	11	7	0.00	0.00
H	P	0	9	0.00	0.00
H	P	0	341	0.00	0.00
H	P	15	199	2.00	2.00
H	P	0	96	0.00	0.00
H	P	0	14	0.00	0.00
H	P	0	0	0.00	0.00
H	P	0	0	0.00	0.00
L	C	0	0		
L	C	348	0	2.29	8.67
L	C	504	0	2.22	14.33
L	C	349	0	1.73	16.33
L	C	27	11	1.28	8.33
L	C	331	0	1.70	16.00
L	C	67	30	2.46	7.67
L	C	100	23	1.77	8.67
L	C	155	0	2.13	8.67
L	C	136	0	2.20	7.67
L	C	116	0	2.06	11.00
L	C	115	0	2.04	7.67
L	P	0	0	1.99	10.67
L	P	0	0		
L	P	0	0		
L	P	0	3		
L	P	5	0		
L	P	268	217	1.94	5.67
L	P	378	289	2.03	4.00

L	P	5	569	2.00	0.67
L	P	0	534	0.00	0.00
L	P	8	42	0.00	0.00
L	P	39	342	1.86	4.33
L	P	343	93	2.41	11.33
L	P	121	0	2.55	7.33

Table 3: For the crab eating trial; by oxygen treatment, data on crab length and weight, as well as speed of consumption.

Oxygen treatment (High/Low)	Crab length (mm)	Crab weight (g)	Consumption speed (g/s)
H	45	12.77	0.0017
H	45	15.82	0.0029
L	45	13.4	0.0027
L	45	14.08	0.0022
L	45	22.71	0.0027
L	37	14.74	0.0009
H	39	13.98	0.0027
H	40	15.81	0.0029
L	45	15.01	0.0020
L	45	17.55	0.0027
L	36	9.21	0.0012
L	40	13.56	0.0028
H	40	12.92	0.0010
H	40	15.32	0.0027
H	40	16.87	0.0031
H	43	15	0.0019
H	43	16.28	0.0020
H	40	13.3	0.0038
L	35	8.76	0.0018
H	43	14.79	0.0046
H	37	9.17	0.0018
L	38	10.82	0.0020
L	40	13.19	0.0032
H	45	20.64	0.0013
L	37	11.24	0.0018
L	38	10.98	0.0022
H	37	12.48	0.0027
L	40	12.82	0.0016
H	42	14.22	0.0049
L	42	15.38	0.0054
L	40	13.28	0.0010
L	45	19.58	0.0038
L	38	10.8	0.0019
H	35	8.81	0.0018
H	39	11.14	0.0025
L	45	21.7	0.0043
L	44	16.63	0.0025
H	37	9.98	0.0031
H	39	12.64	0.0009
H	37	11.89	0.0008
